# Improved Differential Evolution Algorithm Guided by Best and Worst Positions Exploration Dynamics

**DOI:** 10.3390/biomimetics9020119

**Published:** 2024-02-16

**Authors:** Pravesh Kumar, Musrrat Ali

**Affiliations:** 1 ASH (Mathematics) Department, REC Bijnor, Chandpur 246725, UP, India; praveshtomariitr@gmail.com; 2 Department of Basic Sciences, Preparatory Year, King Faisal University, Al Ahsa 31982, Saudi Arabia

**Keywords:** optimization, differential evolution, meta-heuristics, crossover

## Abstract

The exploration of premium and new locations is regarded as a fundamental function of every evolutionary algorithm. This is achieved using the crossover and mutation stages of the differential evolution (DE) method. A best-and-worst position-guided novel exploration approach for the DE algorithm is provided in this study. The proposed version, known as “Improved DE with Best and Worst positions (IDEBW)”, offers a more advantageous alternative for exploring new locations, either proceeding directly towards the best location or evacuating the worst location. The performance of the proposed IDEBW is investigated and compared with other DE variants and meta-heuristics algorithms based on 42 benchmark functions, including 13 classical and 29 non-traditional IEEE CEC-2017 test functions and 3 real-life applications of the IEEE CEC-2011 test suite. The results prove that the proposed approach successfully completes its task and makes the DE algorithm more efficient.

## 1. Introduction

Nowadays, the optimization problems of various science and engineering domains are becoming more complex due to the presence of various algorithmic properties like differentiability, non-convexity, non-linearity, etc., and hence it is not possible to deal with them using traditional methods. For that reason, new meta-heuristic methods are emerging to deal with these challenges in optimization fields. A meta-heuristic is a general term for heuristic methods that can be useful in a wider range of situations than the precise conditions of any specific problem. These meta-heuristic methods can be categorized into different groups, such as (i) the EA-based group, e.g., genetic algorithm [1], differential evolution algorithm [2], Jaya algorithm [3], etc.; (ii) swarm-based group, e.g., particle swarm optimization [4], artificial bee colony [5], gray wolf optimization [6], whale optimization algorithm [7], manta ray foraging optimization [8], reptile search algorithm [9], etc.; (iii) physics-based group, e.g., gravitational search algorithm [10], sine-cosine algorithm [11], atom search optimization [12], etc.; and (iv) human-based group, e.g., brain storm optimization [13], teaching–learning-based optimization [14], gaining–sharing knowledge optimization [15], etc.

The DE algorithm has maintained its influence for the last three decades due to its excellent performance. Many of its variants have placed among the top ranks in the IEEE CEC conference series [16,17]. Its straight forward execution, simple and small structure, and quick convergence can be considered the main reasons for its great efficiency. It has been successfully applied to a wide range of real-life applications, such as image processing [18,19], industriel noise recognition [20], bit coin price forecasting [21], optimal power flow [22], neural network optimization [23], engineering design problems [24], and so on. There are also several other fields like controlling theory [25,26] which are also open for the application of the DE algorithm.

In spite of its many promising characteristics, DE also faces some shortcomings, such as stagnation problems, a slow convergence rate, and a failure to perform in many other critical situations. In the past three decades, a number of studies have been executed to improve its performance and overcome its shortcomings. Many improvements have been developed in the areas of mutation operation and control parameter adjustment. For example, Brest et al. [27] suggested a self-adaptive method of selecting control parameters F and Cr. Later, Zhang et al. [28] proposed JADE by adapting Cauchy distributed control parameters and the *DE/current to p-best/2* strategy. Gong et al. [29] made self-adaptive rules to implement various mutation strategies with JADE. The idea behind JADE was further improved in SHADE [30] by maintaining a successful history memory of the control parameters. Later, LSHADE [31] was proposed to improve the search capacity of SHADE by adapting a linear population size reduction approach. Later, several enhanced variants, such as iLSHADE [32], LSHADE-SPA [33], LSHADE-CLM [34], and iLSHADE-RSP [35], were also presented to improve the performance of the LSHADE variant. The iLSHADE variant was also improved by Brest et al. in their new variant named jSO [36]

Despite these famous variants, there are many other DE variants that have been presented throughout the years, for which some diverse tactics have been adapted to modify the operation of mutation; for example, Ali et al. applied a Cauchy distribution-based mutation operation and proposed MDE [37]. Later, Choi et al. [38] modified the MDE and presented ACM-DE by adapting the advanced Cauchy mutation operator. Kumar and Pant presented MRLDE [39] by dividing the population into three sub regions in order to perform mutation operations. Mallipeddi et al. presented EPSDE [40] using ensemble mutation strategies. Gong and Cai [41] introduced a ranking-based selection idea of using vectors for mutation operation in the current population. Xiang et al. [42] combined two mutation strategies, *DE/current/1/bin* and *DE/p-best/bin/1*, to enhance the performance of the DE algorithm. Some recent research on the development of mutation operations is included in [43,44,45,46,47,48,49].

Apart from these, several good research projects have also been executed in different domains, such as improving population initializing strategies [50,51,52,53], crossover operations [54], selection operations [55,56], local exploration strategies [57,58,59], and so on.

An interesting and detailed literature survey on modifications in the DE algorithm over the last decades is given in [60].

It can be noticed that most of the advanced DE variants compromise their simple structure by including some supplementary features. Therefore, in order to enhance the performance of the DE algorithm without overly complicating its simple structure, a new exploration method guided by the best and worst positions is proposed in this paper. The proposed method attempts to optimally explore the search space by moving forward toward the best position or backward toward the worst position. Additionally, a DE/α_best_/1 [39,42] approach is also incorporated with the proposed exploration strategies in the selection operation to achieve a better balance between exploitation and exploration. The proposed variant is termed as ‘IDEBW’ and has been implemented in various test cases and real-life applications.

The remaining of the paper is designed as follows: a concise description of DE is given in Section 2. The proposed approach for IDEBW variant is explained in Section 3. The parameter settings and the empirical results from various test suites and real-life applications are discussed in Section 4. Finally, the conclusion of the complete study is presented in Section 5.

## 2. DE Algorithm

A basic representation of DE can be expressed as *DE/a/b/c*, where ‘*a*’ *stands for* a mutation approach, ‘*b*’ *stands for* vector differences, and ‘*c*’ *stands for* a crossover approach. The various phases in the operation of the DE algorithm are explained next.

The working structure of the DE algorithm is very easy to implement. It begins with a random generated population $Pop^{\left(G\right)}=\{Y_{i}^{\left(G\right)}\left|i=1,\;2,\;\cdots ,\;N\}\right.$ of *d*-dimensional *N*-vectors within a specified bound domain [*Y_l_*, *Y_u_*], as shown in Equation (1).

(1) $$Y_{i}^{\left(G\right)}=rand\times (Y_{u}-Y_{l})+Y_{l}$$

Subsequently, the mutation, crossover, and selection phases are started for the generation and selection of new vectors for the next-generation population.

*Mutation*: This phase is considered as a key operation in the DE algorithm and can be used to explore new positions in the search space. Some mutation schemes to generate a perturbed vector, say, $M_{i}^{\left(G+1\right)}=\{m_{i,\;j}^{\left(G+1\right)}:\;j=1,\;2,\ldots d\}$, are given in Equation (2).

(2) $$\begin{array}{l} \mathrm{DE}/\;\mathrm{rand}/\;1:\;\;\;\;\;\;\;\;M_{i}^{\left(G+1\right)}=Y_{a_{1}}^{\left(G\right)}+F\times (Y_{a_{2}}^{\left(G\right)}-Y_{a_{3}}^{\left(G\right)})\\ \mathrm{DE}/\;\mathrm{rand}/\;2:\;\;\;\;\;\;\;\;M_{i}^{\left(G+1\right)}=Y_{a_{1}}^{\left(G\right)}+F\times (Y_{a_{2}}^{\left(G\right)}-Y_{a_{3}}^{\left(G\right)})+F\times (Y_{a_{3}}^{\left(G\right)}-Y_{a_{4}}^{\left(G\right)})\\ \mathrm{DE}/\;\mathrm{best}/\;1:\;\;\;\;\;\;\;\;M_{i}^{\left(G+1\right)}=Y_{best}^{\left(G\right)}+F\times (Y_{a_{2}}^{\left(G\right)}-Y_{a_{3}}^{\left(G\right)})\\ \mathrm{DE}/\;\mathrm{best}/\;2:\;\;\;\;\;\;\;\;M_{i}^{\left(G+1\right)}=Y_{best}^{\left(G\right)}+F\times (Y_{a_{2}}^{\left(G\right)}-Y_{a_{3}}^{\left(G\right)})+F\times (Y_{a_{3}}^{\left(G\right)}-Y_{a_{4}}^{\left(G\right)})\\ \mathrm{DE}/\;\mathrm{curr}\;-\mathrm{best}/1:\;M_{i}^{\left(G+1\right)}=Y_{i}^{\left(G\right)}+F\times (Y_{best}^{\left(G\right)}-Y_{i}^{\left(G\right)})+F\times (Y_{a_{2}}^{\left(G\right)}-Y_{a_{3}}^{\left(G\right)}) \end{array}$$

where $Y_{a_{1}},\;Y_{a_{2}}\;Y_{a_{3}},\;Y_{a_{4}}\;Y_{a_{5}}$ are mutually different vectors randomly chosen from $P^{\left(g\right)}$; the parameter F∈(0, 1] is used to manage the magnification of the vector’s difference.

*Crossover:* This phase is generally responsible for maintaining the population diversity and generates a trail vector $X_{i}^{\left(G+1\right)}=\{x_{i,\;j}^{\left(G+1\right)}:\;j=1,\;2,\ldots d\}$ by blending the target $Y_{i}^{\left(G+1\right)}=\{y_{i,\;j}^{\left(G+1\right)}:\;j=1,\;2,\ldots d\}$ and perturbed vector $M_{i}^{\left(G+1\right)}=\{m_{i,\;j}^{\left(G+1\right)}:\;j=1,\;2,\ldots d\}$, as explained in Equation (3).

(3) $$x_{i,\;j}^{\left(g+1\right)}=\left\{\begin{array}{c} m_{i,\;j}^{\left(G+1\right)}\;if\;rand\leq C_{R}\;||\;j\in randi(d)\\ y_{i,\;j}^{\left(G\right)}\;otherwise \end{array}\right.$$

where $C_{R}\in (0,\;1)$ isknown as the crossover parameter, and *randi (D)* denotes the random index used to ensure that at least one component in the trail vector is chosen from the mutant vector.

*Selection*: This procedure selects the best vector from the target and trail vectors for the next-generation population based on their fitness value, as determined by Equation (4).

(4) $$Y_{i}^{\left(g+1\right)}=\left\{\begin{array}{c} X_{i}^{\left(g+1\right)}\;if\;fun\;(X_{i}^{\left(g+1\right)})\leq fun(Y_{i}^{\left(g\right)})\\ Y_{i}^{\left(g\right)}\;else \end{array}\right.$$

## 3. Proposed IDEBW Algorithm

To improve the performance of the DE algorithm without making any major changes to its structure, we designed our variant IDEBW by modifying the original DE algorithm in two ways. We did this by first exploring the search area, guided by best and worst positions, and second by improving the selection operation, where a DE/α_best_/1 approach is also incorporated to generate new trail vectors whenever the old trail vectors are not selected into the next generation. The proposed approaches are explained in detail as below:

### 3.1. Proposed Exploration Strategies

Rao [3] presented the idea of searching for new positions by going towards the best position and away from the worst positions, as shown in Equation (5).

(5) $$Y\prime_{i}^{\left(G\right)}=Y_{i,}^{\left(G\right)}+rand\times (Y_{best,}^{\left(G\right)}-\left|Y_{i,}^{\left(G\right)}\right|)-rand\times (Y_{worst,}^{\left(G\right)}-\left|Y_{i,}^{\left(G\right)}\right|)$$

Motivated by this remarkable idea, we have utilized this approach to explore new positions through mutation and crossover phases, as given below.

To find the new position *X_i_* corresponding to the *i*^th^ vector *Y_i_*, first we chose a random vector, say *Y_r_*, from the population and used Equations (6) and (7) to create the component of the *X_i_*:

*Crossover Operation by Best Position:*

(6) $$DE/rand/best/1:\;\;\;x_{i,j}^{\left(G\right)}=\left\{\begin{array}{l} y_{r,j}^{\left(G\right)}+rand_{B}\times (y_{best,j}^{\left(G\right)}-y_{i,j}^{\left(G\right)});\;if\;rand\leq CR_{B}\;\\ y_{i,j}^{\left(G\right)};\;otherwise \end{array}\right.$$

*Crossover Operation by Worst Position:*

(7) $$DE/rand/worst/1:\;\;\;x_{i,j}^{\left(G\right)}=\left\{\begin{array}{l} y_{r,j}^{\left(G\right)}-rand_{W}\times (y_{worst,j}^{\left(G\right)}-y_{i,j}^{\left(G\right)});\;if\;rand\leq CR_{W}\;\\ y_{i,j}^{\left(G\right)};\;otherwise \end{array}\right.$$

where *rand*, *rand_B_* and *rand_W_* are different uniform random numbers from 0 to 1, and *CR_B_* and *CR_W_* are prefix constants used to handle the crossover rate. Now we can randomly pick any proposed crossover strategy on the basis of pre-fix probability, called ‘*P_r_*’.

**Figure 1:**
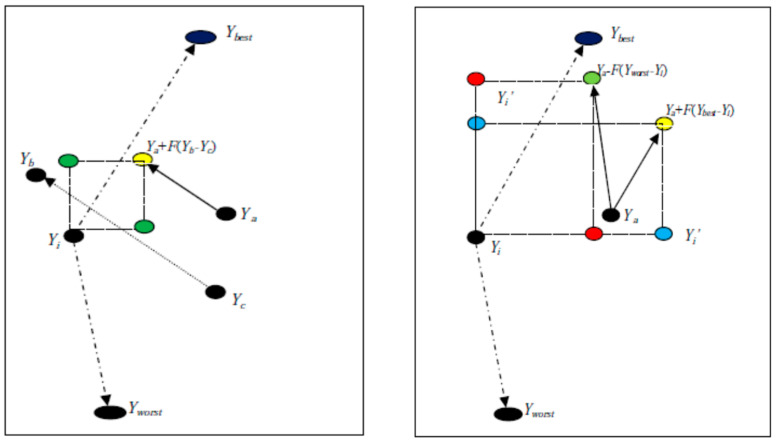
Difference of exploration by DE/rand/mutation and proposed mutation.

The difference between explorations by the *DE/rand/1* and proposed strategies is graphically demonstrated in Figure 1. In the left image, the yellow and green dots represent the possible crossover position as determined using the DE/rand/1 strategy. When using this strategy, we can see that there are four possible crossover positions for the target vector *Y_i_*. In the right image, the yellow and blue dot represent the possible crossover position as assessed using the *DE/rand/best/1* strategy, while the green and red dot represents the possible crossover position as determined using the *DE/rand/worst/1* strategy. We can see that eight improved possible crossover positions for the target vector *Y_i_* are obtained using these strategies. Hence, we can say that the proposed strategies improve the exploration capability of the DE algorithm by providing additional and better positions for generating trail vectors compared to the *DE/rand/1* approach.

### 3.2. Improved Selection Operation

If a vector created through the proposed crossover operation was not able to beat its target vector, then we imposed *DE/α_best_/1* to create an additional trail vector. This approach is an adapted version of *DE/rand/1* and also utilizes the advantage of another approach, namely *DE/best/1*, by selecting the base vector *Y_a_* from the top α% of the current population. The crossover operation for the *DE/α_best_/1* is defined by Equation (8) as below:

(8) $$DE/\alpha_{best}/1:\;x_{i,\;j}^{\left(G+1\right)}=\left\{\begin{array}{l} y_{a_{1}*,\;j}^{\left(G\right)}+F_{\alpha }\times (y_{a_{2},j}^{\left(G\right)}-y_{a_{3},j}^{\left(G\right)});\;\;\;if\;rand\leq CR_{\alpha }||\;j\in randi\;(1,\;2,\;\ldots d)\\ y_{i,\;j}^{\left(G\right)};\;\;otherwise \end{array}\right.$$

where $Y_{a_{1}}$ is a randomly selected vector from the top α% of the current population; $Y_{a_{2}}$ and $Y_{a_{2}}$ are another two randomly selected vectors; and $F_{\alpha }$ and $CR_{\alpha }$ are control parameters.

Therefore, by using the proposed IDEBW, we not only obtain an additional approach to generating the trail vector, but also a way to improve it via a modified selection operation. However, apart from these advantages, we can also face drawbacks like slightly increased complexity and population stagnation problems in some cases.

**Figure 2:**
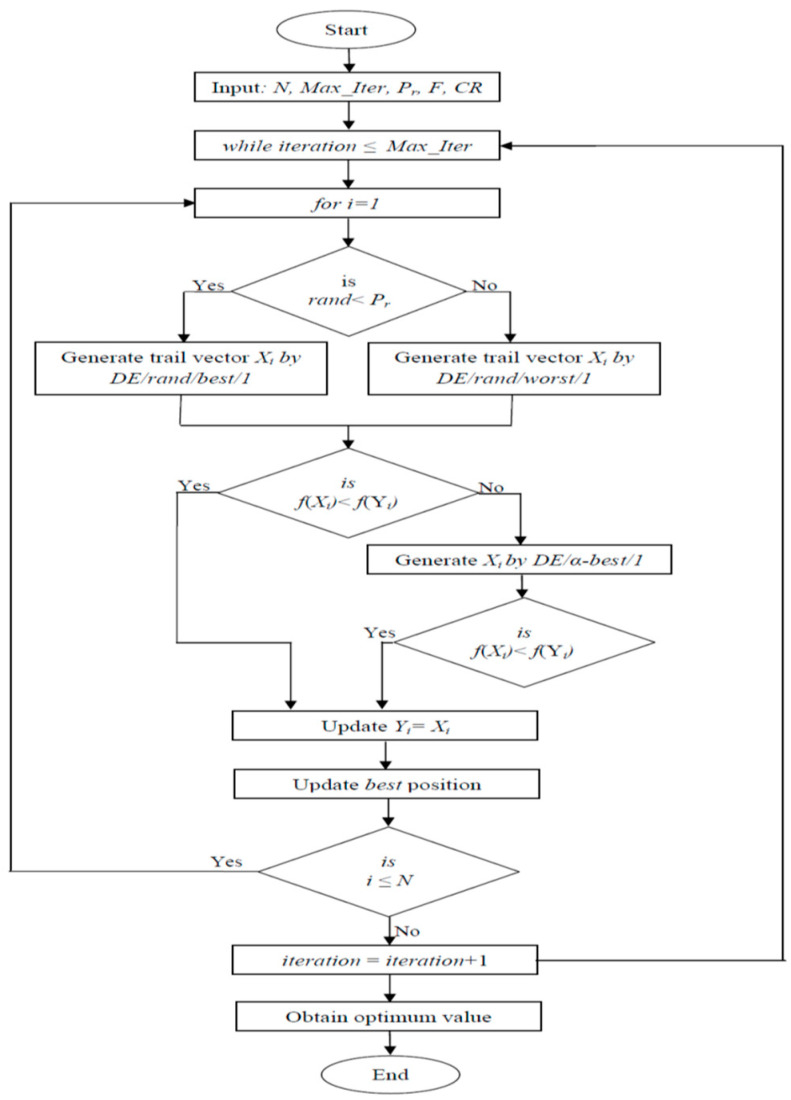
Flow chart of IDEBW.

The working steps, pseudo-code (Algorithm 1), and flowchart (Figure 2) of the proposed IDEBW are given as below:

*(a)*  *Working Steps:*
  *Step-1:*  Initialize the parameter settings, like population size (*N*), *CR_B_*, *CR_W_*, *CR_α_*, *F_α_*, probability constant (*P_r_*), and *Max-iteration*, and generate initial population.
  *Step-2:*  Generate a uniform random number *rand* and go to step-3.
  *Step-3:*  If (*rand* ≤ *P_r_*) then use Equation6; otherwise, use Equation (7) to generate trail vector.
  *Step-4:*  Select this trail vector for the next generation if it gives a smaller fitness value than its corresponding target vector; otherwise, generate an additional trail vector using Equation (8) and repeat the old selection operation.
  *Step-5:*  Repeat all above steps for all remaining vectors and obtain the best value after Max-iteration reached.
*(b)*  *Pseudo-Code of proposed IDEBW***Algorithm 1. IDEBW Algorithm**1**Input:** *N*, *d*, Max-iteration, *CR_B_*,* CR_W_*,* CR_α_*,* F_α_*2$\mathrm{Generate}\;\mathrm{initial}\;\mathrm{population}\;P^{\left(G\right)}$ via Equation (1)3Calculate function value *f*(*Y_i_*)** for each *i*4**While** *iteration ≤ Max_Iteration*5Obtain best and worst locations6**For** *i* = 1:*N*7$\mathrm{Select}\;Y_{r}\;\mathrm{randomly}\;\mathrm{from}\;P^{\left(G\right)}$8**IF** *rand* ≤ *P_r_*9**For** *j* = 1:*d*10$\mathrm{Generate}\;\mathrm{trail}\;\mathrm{vector}\;X_{i}$ via Equation (6)//(*DE/rand/best/1*)11**End For**12**Else**13**For** *j* = 1:*d*14$\mathrm{Generate}\;\mathrm{trail}\;\mathrm{vector}\;X_{i}$ via Equation (7)//(*DE/rand/best/1*)15**End For**16**End IF**17**IF** $f(X_{i})\leq f\;(Y_{i})$18$\mathrm{Update}\;Y_{i}$ $\mathrm{via}\;X_{i}$19Update best position20**Else**21$\mathrm{Select}\;Y_{a_{1}}$ $\mathrm{randomly}\;\mathrm{from}\;\mathrm{top}\;\alpha \%\;\mathrm{and}\;Y_{a_{2}}$$\;\mathrm{and}\;Y_{a_{3}}$$\;\mathrm{from}\;\mathrm{the}\;P^{\left(G\right)}$22**For** *j* = 1:*d*23$\mathrm{Generate}\;\mathrm{trail}\;\mathrm{vector}\;X_{i}$ via Equation (8)//(DE/α-best/1)24**End For**25**IF** $f\;(X_{i})\leq f\;(Y_{i})$26$\mathrm{Update}\;Y_{i}$ $\mathrm{via}\;X_{i}$27Update best position28**End IF**29**End IF**30**End For**31*iteration* = *iteration* + 132**End While**
*(c)*  *Flow Chart of proposed IDEBW*

## 4. Result Analysis and Discussion

The performance assessment of the proposed IDEBW on various test suites and real-life problems is discussed in this section.

### 4.1. Experimental Settings

All experiments are executed under the following conditions:

- System Configuration: OS-64 Bit, Windows-10, Processor: 2.6-GHz Intel Core i3 processor, RAM-8GB.
- *N=100; d=30,*
- *α = 20, F_α_ = 0.5, CR_α_ = 0.9, CR_B_ = 0.9, CR_W_ = 0.5.*
- *Max-iteration = 100 × d.*
- *Total Run = 30.*

### 4.2. Performance Evaluation of IDEBWon Classical Functions

A test suite of 13 simple and classical benchmark problems is selected from different studies [21,22,23]. The functions can be classified as unimodal (*f*_1_–*f*_6_) and multimodal functions (*f*_8_–*f*_13_),or as noisy function *f*_7_. As per the literature, the unimodal and multimodal functions are essential to testing the exploration and convergence effectiveness of the algorithms.

The performance assessment of IDEBW is performed with six other state-of-the-art DE variants, such as jDE [27], JADE [28], ApadapSS-JADE [29], SHADE [30], CJADE [58],and DEGOS [57]. The results for the jDE and JADE are copied from [28], while the results for the APadapSS-JADE are taken from [29]. For the SHADE, CJADE, and DEGOS, the results are obtained by using the code provided by the respective authors on http://toyamaailab.githhub.io/soucedata.html (accessed on 23 July 2023). The numerical results for the average error and standard deviation of 30 independent runs are presented in Table 1.

From Table 1, it is clear that the proposed IDEBW improves the quality of result, obtaining first rank for eight functions, namely *f*_1_, *f*_2_, *f*_3_, *f*_5_, *f*_10_, *f*_11_, *f*_12_, and *f*_13_, and second rank for function *f*_6_. For remaining functions *f*_4_ and *f*_7_, it takes third rank, while for *f*_8_ and *f*_9_, it takes sixth and fifth ranks, respectively. The Ap-AdapSS-JADE obtains first rank in three cases—*f*_6_, *f*_7_, and *f*_11_—whereas SHADE, JADE and jDE obtain first rank for *f*_4_, *f*_9_ and *f*_8_, respectively. The win/loss/tie (w/l/t) represents the pairwise competition which indicates that the IDEBW exceeds the CJADE, DEGOS, SHADE, AdapSS-JADE, JADE, and jDE in 10, 13, 10, 8, 11 and 11 cases, respectively.

To check the time complexity of the algorithm, the average CPU run time is also calculated for the algorithms IDEBW, CJADE, DEGOS, and SHADE. We can see that the CPU times for IDEBW, CJADE, DEGOS, and SHADE are 11.6, 13.2, 11.4 and 12.1 s, respectively. Hence, IDEBW takes less computing time than CJADE and SHADE. The exception is DEGOS, which is better than all algorithms in terms of time complexity.

The signs ‘+’, ‘−‘ and ‘=’ stand for whether the IDEBW is significantly better, worse, or equal, respectively. The *p*-value for pairwise ‘Wilcoxon sign test’ is also presented in the table, verifying the statistical effectiveness of the proposed IDEBW on the others.

The Wilcoxon rank sum test outcomes are listed in Table 2. The results present pairwise ranks, sum of ranks, and *p*-values. The lower rank and higher positive rank sum evidence the effectiveness of the proposed IDEBW over its competitors. However, the *p*-values shows that the IDEBW is significantly better than CJADE, DEGOS, and jDE, while there is no significant difference between the performance of IDEBW, SHADE, APAdapSS-JADE, and JADE.

The Friedman’s rank and critical difference (CD) values obtained through the Bonferroni–Dunn test are presented in Table 3 in order to examine the global difference between the algorithms. The IDEBW obtained the lowest average rank, confirming its significance over others.

**Figure 3:**
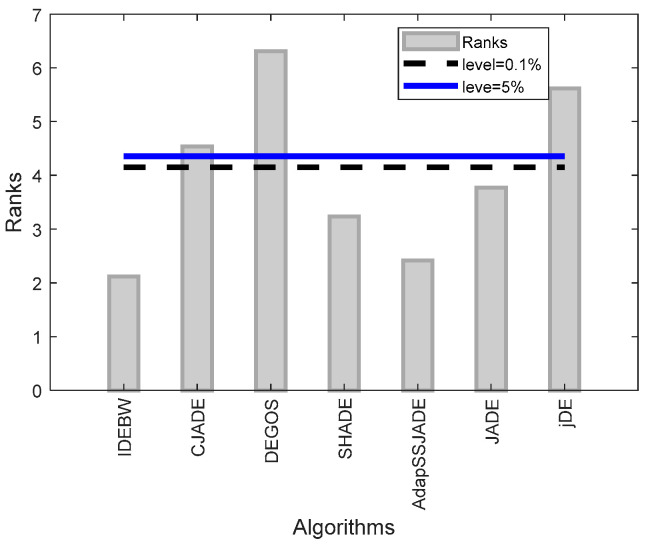
The Friedman ranks and Bonferroni–Dunn test presentation for classical functions.

Figure 3 represents the algorithm’s ranks and horizontal control lines. These show significant levels at 10% and 5%, respectively. Through the graph, we can see that the rank bars of the IDEBW, SHADE, ApadapSS-JADE, and JADE are below the control lines and hence these algorithms are of equal significance, while the CJADE, DEGOS, and jDE are considered significantly worse than the obtained IDEBW algorithm.

**Figure 4:**
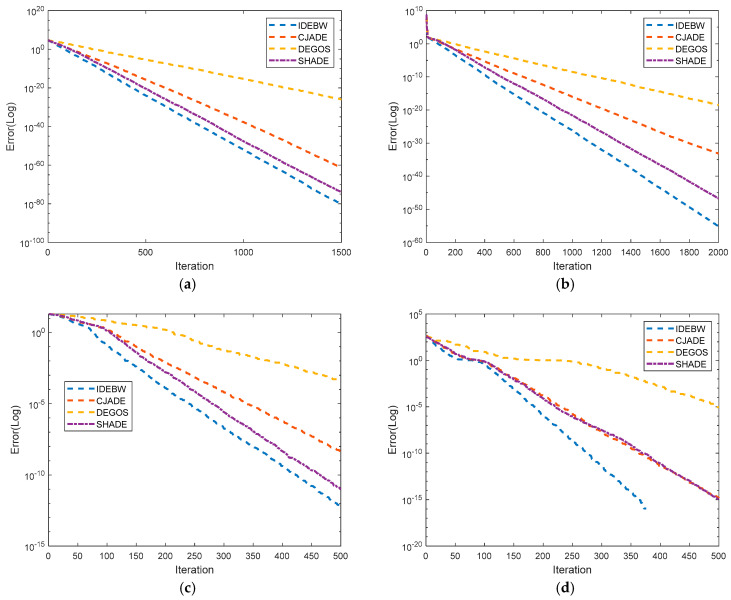
Performance evaluation of IDEBW by convergence graphs of classical functions. (**a**) F_01_ (Unimodal). (**b**) F_02_ (Unimodal). (**c**) F_10_ (Multimodal). (**d**) F_11_ (Multimodal).

Figure 4 represents the convergence graphs of the algorithms for some selected functions: *f*_1_, *f*_2_, *f*_10_ and *f*_11_. The X- and Y-axes indicate the iterations and fitness values of the function. We can analyze the convergence behaviour of the algorithms using their graph lines, which verifies the faster convergence of the proposed IDEBW than its competitors.

### 4.3. Performance Evaluation of IDEBW on CEC2017 Functions

In this section, a performance assessment of the IDEBW is performed on a well-known IEEE CEC-2017 test suite of 29 (*C*_1_–*C*_30_) more complicated and composite functions. These functions can be divided into four groups: unimodal (*C*_1_–*C*_3_), multimodal (*C*_4_–*C*_10_), hybrid (*C*_11_–*C*_20_), and composite (*C*_21_–*C*_30_). For a function, the optimum value is 100×function_no, while the initial bounds are (−100, 100) for all functions. A full specification of these functions is given in [61].

Next the performance Assessment of IDEBW with DE Variants and other meta-heuristics have been carried out separately and their numerical results are presented in Table 4 and Table 5 respectively while the statistical analysis on these results are given in Table 6 and Table 7.

#### 4.3.1. Performance Assessment with DE Variants

Five state-of-the-art DE variants, such as SHADE [30], DEGOS [57], CJADE [58], TRADE [59] and IMODE [62] are selected for performance assessment with IDEBW. The TRADE, CJADE, and DEGOS are recently developed DE variants, while the SHADE and IMODE are the winner algorithms from the CEC-2014 and CEC-2020 competitions, respectively. The population size and maximum iterations are taken as 100 and 3000, respectively, for all algorithms. The other parameter settings of algorithms are taken as suggested in their original works.

Table 4 presents the numerical results for the average error and standard deviation of 30 runs. The value to reach (VTR) is taken as 10^−08^, i.e., the error is taken as 0 if it crosses the fixed VTR. Table 4 shows that the IDEBW obtains first rank in 11 cases, such as *C*_1_, *C*_6_, *C*_9_, *C*_13_, *C*_15_, *C*_18_, *C*_19_, *C*_22_, *C*_25_, *C*_29_ and *C*_30_. Similarly, TRADE obtains first rank in 11 cases, such as *C*_1_, *C*_6_, *C*_9_, *C*_16_, *C*_17_, *C*_20_, *C*_22_, *C*_23_, *C*_25_, *C*_26_ and *C*_27_. SHADE obtains best position in 10 cases, such as *C*_1_, *C*_3,_ *C*_5_, *C*_7_, *C*_8_, *C*_10_, *C*_21_, *C*_22_, *C*_24_, and *C*_25_. The CJADE and DEGOS both obtain first ranks in 5 cases such as (*C*_1_, *C*_6_, *C*_9_, *C*_22_, and *C*_25_) and (*C*_1_, *C*_9_, *C*_11_, *C*_14_, and *C*_22_), respectively, whereas IMODE takes first place in only 3 cases, such as *C*_4_, *C*_12_, and *C*_28_. All algorithms except IMODE equally obtain first rank for *C*_1_ and *C*_22_, while the IDEBW, TRADE, DEGOS and CJADE perform equally in the case of *C*_6_ and *C*_9_. The pairwise w/l/t performance demonstrates that the IDEBW exceeds the TRADE, CJADE, DEGOS, SHADE, and IMODE in 13, 14, 16, 14 and 25 cases, respectively.

The average CPU times for the IDEBW, TRADE, CJADE, DEGOS, SHADE and IMODE are 146.2, 165.4, 172.9, 144.5, 148.1, and 168.2 s, respectively. Hence, IDEBW takes less computing time than all DE variants except DEGOS, which is better than all algorithms in term of time complexity.

The *p*-values obtained by the pairwise ‘Wilcoxon sign test’ also verify the statistical effectiveness of the proposed IDEBW on the others.

The Wilcoxon rank sum test outcomes with pairwise ranks, sum of ranks, and *p*-values are listed in Table 6. The lower rank and higher positive rank sum evidence the effectiveness of the proposed IDEBW over its competitors. However, the *p*-values show that the IDEBW is significantly better than IMODE, while there is no significant difference between the performance of the IDEBW, TRADE, CJADE, DEGOS, and SHADE.

**Figure 5:**
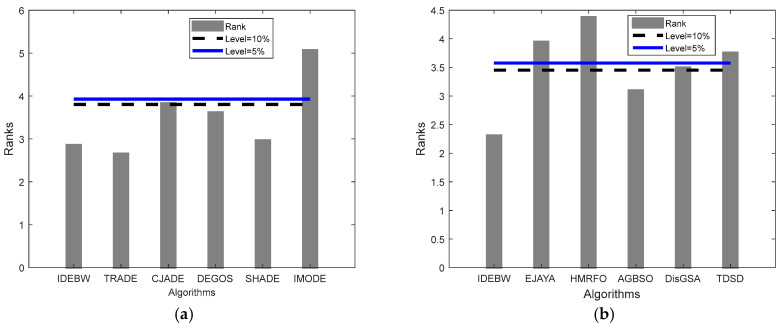
The Friedman ranks and Bonferroni–Dunn test presentation for CEC17 functions for (**a**) DE varaints. (**b**) Meta-hueristics varaints

The Friedman’s rank and critical difference (CD) values obtained through the Bonferroni–Dunn test are presented in Table 7 to test out the global difference between the algorithms. The TRADE obtained lowest average rank; however, the bar graphs presented in Figure 5a shows that the IDEBW, TRADE, DEGOS, and SHADE are considered as significantly equal, while the CJADE and IMODE are significantly worse with these algorithms.

#### 4.3.2. Performance Assessment with Other Meta-Heuristics

In this section, the performance of the IDEBW is compared with that of 5 other meta-heuristics algorithms such as TDSD [63], EJaya [64], AGBSO [65], HMRFO [66],and disGSA [67]. The HMRFO, disGSA, AGBSO, and EJaya methods are recently developed variants of meta-heuristics such as MRFO, GSA, BSO, and Jaya algorithms, respectively, whereas the TDSD is a hybrid variant of three search dynamics such as spherical search, hypercube search, and chaotic local search.

The population size and maximum iterations are taken as 100 and 3000, respectively, for all algorithms. The other parameter settings of algorithms are taken as suggested in their original works.

Table 5 presents the obtained average error and standard deviation of 30 runs. The Table 5 shows that IDEBW obtains first rank in 14 cases, namely, *C*_1_, *C*_6_, *C*_9_, *C*_11_, *C*_13_, *C*_14_, *C*_15_, *C*_18_, *C*_19,_ *C*_20_, *C*_22_, *C*_27_, *C*_29_,and *C*_30_, whereas AGBSO obtains first rank in 9 cases *C*_5_, *C*_8_, *C*_9_, *C*_10_, *C*_16_, *C*_17_, *C*_21_, *C*_22_, and *C*_23_. The EJAYA, HMRFO, disGSA, and TDSD obtain first ranks in 3 cases (*C*_3_, *C*_12_, and *C*_22_), 1 case (*C*_22_), 4 cases (*C*_7_, *C*_22_, *C*_24_, and *C*_26_), and 2 cases (*C*_4_, *C*_25_), respectively. The pairwise w/l/t demonstrates that the IDEBW exceeds the EJAYA, HMRFO, AGBSO, disGSA, and TDSD on 24, 25, 16, 17 and 22 cases, respectively.

The average CPU times for IDEBW, EJAYA, HMRFO, AGBSO, DisGSA, and TDSD are 146.2, 105.4, 165.2, 154.4, 159.2, and 189.3 s, respectively. Hence, IDEBW takes less computing time than all meta-heuristics except EJAYA, which is better than all algorithms in term of time complexity.

The *p*-values, obtained by the pairwise ‘Wilcoxon sign test’, also verify the statistical effectiveness of the proposed IDEBW on the others.

The Wilcoxon rank sum test outcomes with pairwise ranks, sum of ranks, and *p*-values are listed in Table 6. The lower rank and higher positive rank sum evidence the effectiveness of the proposed IDEBW over its competitors. The *p*-values show that only AGBSO demonstrated a significantly equal performance with the IDEBW, whereas all other meta-heuristics are significantly worst against the IDEBW.

The Friedman’s rank and critical difference (CD) values obtained through the Bonferroni–Dunn test are presented in Table 7 to test out the global difference between the algorithms. The IDEBW obtains the lowest average rank and shows its significance.

The bar graphs presented in Figure 5b show that the IDEBW and AGBSO are significantly equal, while the others cross the control lines and are considered as significantly worse compared to those with these algorithms.

**Figure 6:**
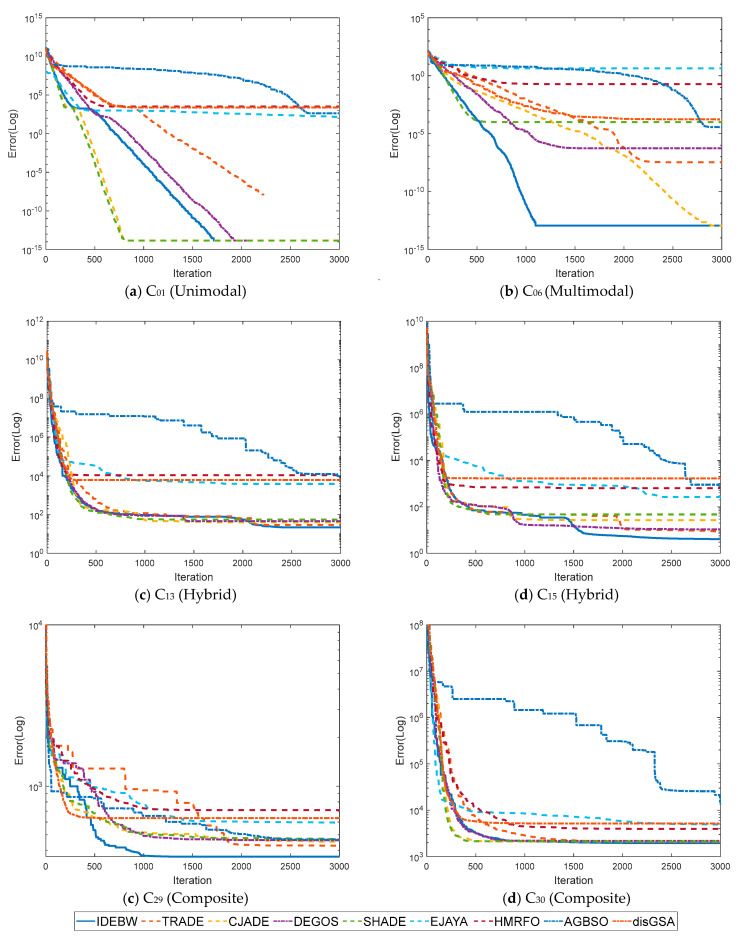
Convergence graphs for CEC-2017 functions: (**a**) *C*_01_, (**b**) *C*_05_, (**c**) *C*_15_, and (**d**) *C*_30._

Figure 6 represents the convergence graphs of the algorithms for some selected functions: *C*_1_, *C*_10_, *C*_21_, and *C*_30_. The X and Y-axes indicate the iterations and fitness values of the function. We can analyze the convergence behaviour of the algorithms by their graphs lines, which verify the faster convergence of the proposed IDEBW on its competitors.

### 4.4. Performance Evaluation of IDEBW on Real-Life Applications

In this section, the practical qualification of the proposed IDEBW is tested on 03 IEEE CEC-2011 real-life applications, as given below:

RP_1_: Frequency-modulated (FM) sound wave problem.
RP_2_: Spread-spectrum radar polyphase code design problem.
RP_3_: Non-linear stirred tank reactor optimal control problem.

The complete details of these problems are specified in [68].

The performance assessment is taken with five qualified algorithms, including DEGOS, SHADE, DE, EJAYA, and TDSD. The outcomes for the SHADE and TDSD are copied from [63]. The maximum iterations are taken as 100 × *d*, i.e., it is 600, 2000, and 100 for the RP_1_, RP_2_, and RP_3_ respectively. The results for the best values, mean values and standard deviation obtained in 30 independent runs are presented in Table 8.

The results show that the proposed IDEBW improves the quality of results and obtains first rank by obtaining the optimum value in each case, whether it is RP_1_, RP_2_,and RP_3_. The SHADE algorithm takes second rank for RP_1_ and RP_2_, while TDSD takes second rank for RP_3_. Hence, the proposed IDEBW confirms its feasibility for use on the real-life problems also.

**Figure 7:**
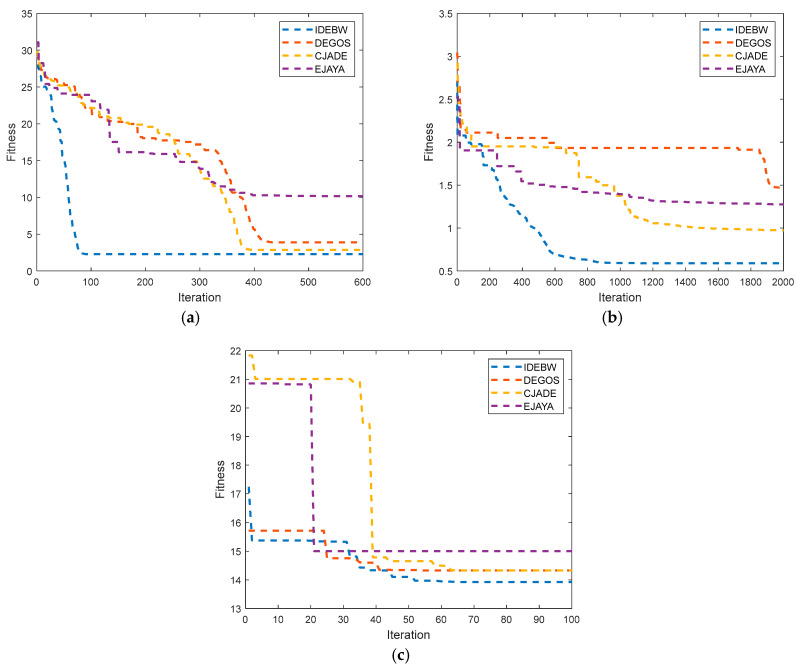
Convergence graphs for real-life problems: (**a**) *RP*_1_ (**b**) *RP*_2_ and (**c**) *RP*_3._

The convergence graphs for the IDEBW, DEGOS, DE, and EJAYA are presented in Figure 7. The X- and Y-axes indicate the iterations and fitness values of the function. We can analyze the convergence behaviour of the algorithms by their graph lines, which also demonstrate a faster convergence speed of the IDEBW compared to its opponents.

## 5. Conclusions

A best and worst location guided exploration approach to the DE algorithm is presented in this study. The proposed technique offers an improved search alternative by either directing attention towards the best location or avoiding the most unfavorable location. The proposed variant named ‘IDEBW’ also uses the DE/α_best_/1 approach as a selection operation when the trail vectors are not selected for the next operation. The ‘IDEBW’ variant is tested on 13 classical, 29 hybrids, and composite CEC-17 benchmark functions and 3 real-life optimization problems from the CEC-2011 test suite. The results are compared with eight other state-of-the-art DE variants, such as jDE, JADE, SHADE, APadapSS-JADE, CJADE, DEGOS, TRADE, and IMODE, and 5 other enhanced meta-heuristics variants, such as EJAYA, HMRFO, disGSA, AGBSO, and TDSD. The outcomes verify the success of the new exploration strategy in terms of improvement in solution quality, as well as in convergence speed.

Our future works will focus on employing the proposed IDEBW in some complicated, constrained, and multi-objective real-life applications. Second, it will also be quite exciting to apply the proposed idea to other meta-heuristic algorithms to improve their performance.

## Figures and Tables

**Table 1:** Performance Evaluation of IDEBW Classical Functions.

F	Iter.	IDEBW	CJADE	DEGOS	SHADE	APadapSS-JADE	JADE	jDE
*f*_1_	1.5 × 10^3^	**3.51 × 10^−81^** **(7.1 × 10^−81^)**	4.07 × 10^−62 +^ (2.32 × 10^−62^)	6.14 × 10^−26 +^ (4.85 × 10^−26^)	3.76 × 10^−74 +^ (2.34 × 10^−74^)	2.45 × 10^−75 +^ (1.39 × 10^−74^)	1.79 × 10^−60 +^ (8.29 × 10^−60^)	2.49 × 10^−28 +^ (4.39 × 10^−28^)
	rank	**1**	4	7	3	2	5	6
*f*_2_	2.0 × 10^3^	**7.08 × 10^−56^** **(4.55 × 10^−56^)**	7.03 × 10^−34 +^ (4.53 × 10^−34^)	1.98 × 10^−19 +^ (3.43 × 10^−19^)	1.04 × 10^−47 +^ (3.24 × 10^−47^)	1.90 × 10^−44 +^ (1.29 × 10^−43^)	1.89 × 10^−25 +^ (9.01 × 10^−25^)	1.49 × 10^−23 +^ (1.01 × 10^−23^)
	rank	**1**	4	7	2	3	5	6
*f*_3_	5.0 × 10^3^	**1.57 × 10^−68^** **(2.19 × 10^−68^)**	1.06 × 10^−59 +^ (9.05 × 10^−59^)	1.39 × 10^−20 +^ (1.09 × 10^−20^)	4.56 × 10^−63 +^ (2.15 × 10^−63^)	2.49 × 10^−68 +^ (8.40 × 10^−68^)	5.99 × 10^−61 +^ (2.90 × 10^−60^)	5.19 × 10^−14 +^ (1.11 × 10^−14^)
	rank	**1**	5	6	3	2	4	7
*f*_4_	5.0 × 10^3^	1.11 × 10^−49^ (1.53 × 10^−49^)	1.97 × 10^−61 +^ (2.34 × 10^−60^)	2.34 × 10^−01 +^ (4.82 × 10^−01^)	**7.86 × 10^−64 −^** **(4.83 × 10^−64^)**	5.15 × 10^−22 +^ (5.39 × 10^−22^)	8.19 × 10^−24 +^ (4.01 × 10^−23^)	1.39 × 10^−15 +^ (1.09 × 10^−15^)
	rank	3	2	7	**1**	5	4	6
*f*_5_	5.0 × 10^3^	**2.14 × 10^−28^** **(1.98 × 10^−28^)**	6.02 × 10^−01 +^ (4.82 × 10^−01^)	9.53 × 10^−22 +^ (4.28 × 10^−22^)	8.12 × 10^−02 +^ (4.34 × 10^−02^)	3.20 × 10^−01 +^ (1.09 × 10^+00^)	8.01 × 10^−02 +^ (7.19 × 10^−01^	1.30 × 10^+01 +^ (1.40 × 10^+01^)
	rank	**1**	6	2	4	5	3	7
*f*_6_	1.0 × 10^2^	1.02 × 10^−01^ (3.22 × 10^−01^)	3.57 × 10^+00 +^ (6.43 × 10^−01^)	9.34 × 10^+01 +^ (3.45 × 10^+01^)	4.11 × 10^+00 +^ (1.01 × 10^+00^)	**3.99 × 10^−02 −^** **(1.95 × 10^−02^)**	2.90 × 10^+00 +^ (1.10 × 10^+00^)	1.09 × 10^+03 +^ (2.09 × 10^+02^)
		2	4	6	5	**1**	3	7
*f*_7_	3.0 × 10^3^	1.05 × 10^−03^ (9.23 × 10^−04^)	1.21 × 10^−03 +^ (5.24 × 10^−03^)	2.22 × 10^−03 +^ (3.34 × 10^−03^)	1.18 × 10^−03 +^ (3.38 × 10^−04^)	**5.89 × 10^−04 +^** **(1.79 × 10^−04^)**	6.39 × 10^−04 −^ (2.19 × 10^−04^)	3.29 × 10^−03 +^ (8.49 × 10^−04^)
	rank	3	5	6	4	**1**	2	**7**
*f*_8_	1.0 × 10^3^	9.49 × 10^02^ (3.37 × 10^02^)	1.05 × 10^−03 −^ (1.39 × 10^−05^)	2.62 × 10^03 +^ (7.11 × 10^03^)	1.01 × 10^−03 −^ (0.00 × 10^00^)	1.79 × 10^−08 +^ (1.20 × 10^−07^)	3.29 × 10^−05 −^ (2.1 × 10^−05^)	**7.19 × 10^−11 −^** **(1.29 × 10^−10^**)
		6	5	7	4	2	3	**1**
*f*_9_	1.0 × 10^3^	1.42 × 10^01^ (2.59 × 10^00^)	7.01 × 10^02+^ (3.22 × 10^00^)	2.53 × 10^01 +^ (1.03 × 10^01^)	3.38 × 10^00 −^ (1.37 × 10^00^)	2.89 × 10^−01 −^ (5.70 × 10^−01^)	**1.09 × 10^−04 −^** **(6.09 × 10^−05^)**	1.49 × 10^−04 −^ (1.99 × 10^−04^)
	rank	5	7	6	4	3	**1**	2
*f*_10_	5.0 × 10^2^	**5.63 × 10^−13^** **(2.81 × 10^−13^)**	4.69 × 10^−09 +^ (3.42 × 10^−09^)	4.85 × 10^−04 +^ (1.09 × 10^−04^)	1.25 × 10^−11 +^ (3.45 × 10^−11^)	1.11 × 10^−11 +^ (1.90 × 10^−10^)	8.19 × 10^−10 +^ (7.01 × 10^−10^)	3.49 × 10^−04 −^ (1.05 × 10^−04^)
	rank	**1**	5	7	3	2	4	6
*f*_11_	5.0 × 10^2^	**0.00** **(0.00)**	1.70 × 10^−15 +^ (4.34 × 10^−16^)	3.33 × 10^−05 +^ (5.32 × 10^−05^)	1.55 × 10^−16 +^ (3.47 × 10^−16^)	**0.00 ^=^** **(0.00)**	9.89 × 10^−08 +^ (6.01 × 10^−07^)	1.89 × 10^−05 +^ (5.79 × 10^−05^)
		**1**	4	6	3	**1**	5	7
*f*_12_	5.0 × 10^2^	**2.13 × 10^−25^** **(1.88 × 10^−25^)**	3.42 × 10^−18 +^ (3.41 × 10^−18^)	5.63 × 10^−04 +^ (8.45 × 10^−04^)	4.56 × 10^−19 +^ (3.23 × 10^−19^)	2.19 × 10^−22 +^ (7.69 × 10^−22^)	4.39 × 10^−17 +^ (2.10 × 10^−16^)	1.59 × 10^−07 +^ (1.50 × 10^−07^)
	rank	**1**	4	7	3	2	5	6
*f*_13_	5.0 × 10^2^	**1.83 × 10^−23^** **(3.47 × 10^−23^)**	4.56 × 10^−17 +^ (4.21 × 10^−17^)	1.23 × 10^−03 +^ (3.42 × 10^−03^)	2.67 × 10^−18 +^ (1.03 × 10^−18^)	3.80 × 10^−20 +^ (1.19 × 10^−19^)	2.09 × 10^−16 +^ (6.59 × 10^−16^)	1.48 × 10^−06 +^ (9.80 × 10^−07^)
	rank	**1**	4	7	3	**2**	5	6
CPU Time (s)		11.6	13.2	11.4	12.1	--	--	--
*w/l/t*			11/2/0 0.022 ^+^	13/0/0 <0.001 ^+^	10/3/0 0.092 ^=^	8/4/1 0.388 ^=^	10/3/0 *p* = 0.092 ^=^	11/2/0 *p* = 0.022 ^+^

‘+’, ‘−‘ and ‘=’ stand for significantly better, worst and equal, respectively.

**Table 2:** ‘Wilcoxon rank sum test’ outcomes for the classical functions.

Algorithms		Pairwise Rank	ΣR^+^	ΣR^−^	z-Value	*p*-Value	Sig at α = 0.05
IDEBW vs.	CJADE	(1.15, 1.85)	75	16	2.062	0.039	+
	DEGOS	(1.00, 2.00)	91	0	3.180	0.001	+
	SHADE	(1.23, 1.77)	63	28	1.223	0.221	=
	APadapSS-JADE	(1.35, 1.65)	40	38	0.078	0.937	=
	JADE	(1.23, 1.77)	57	34	0.804	0.422	=
	jDE	(1.15, 1.85)	75	16	2.062	0.039	+

‘+’, ‘−‘ and ‘=’ stand for significantly better, worst and equal, respectively.

**Table 3:** Friedman Ranks and Bonferroni–Dunn’s CD values for classical functions.

	IDEBW	CJADE	DEGOS	SHADE	ApadapSS-JADE	JADE	jDE	CD (α = 0.1)	CD (α = 0.05)
Rank	2.12	4.54	6.31	3.23	2.42	3.77	5.62	2.0285	2.2352

**Table 4:** Comparison of IDEBW with other DE variants on CEC-2017 functions.

*Fun*	IDEBW		TRADE		CJADE		DEGOS		SHADE		IMODE	
	Mean	SD	Mean	SD	Mean	SD	Mean	SD	Mean	SD	Mean	SD
*C*_1_	0.00 × 10^00^	0.00 × 10^00^	0.00 × 10^00^	0.00 × 10^00^	0.00 × 10^00^	0.00 × 10^00^	0.00 × 10^00^	0.00 × 10^00^	0.00 × 10^00^	0.00 × 10^00^	8.1 × 10^−11^	1.4 × 10^−03^
*C*_3_	1.8 × 10^−08^	1.9 × 10^−07^	2.40 × 10^01^	4.41 × 10^01^	8.5 × 10^−04^	1.42 × 10^04^	2.8 × 10^−05^	6.9 × 10^−05^	0.00 × 10^00^	0.00 × 10^00^	1.4 × 10^−07^	8.1 × 10^−09^
*C*_4_	5.86 × 10^01^	0.00 × 10^00^	5.98 × 10^01^	2.45 × 10^00^	3.66 × 10^01^	3.08 × 10^01^	5.92 × 10^01^	1.85 × 10^00^	5.86 × 10^01^	3.1 × 10^−14^	2.19 × 10^01^	2.84 × 10^02^
*C*_5_	3.55 × 10^01^	1.22 × 10^01^	1.90 × 10^01^	4.91 × 10^00^	2.66 × 10^01^	6.09 × 10^00^	2.70 × 10^01^	1.25 × 10^01^	1.55 × 10^01^	2.70 × 10^00^	2.59 × 10^02^	4.14 × 10^00^
*C*_6_	0.00 × 10^00^	0.00 × 10^00^	0.00 × 10^00^	0.00 × 10^00^	0.00 × 10^00^	0.00 × 10^00^	7.9 × 10^−07^	1.5 × 10^−06^	3.8 × 10^−05^	3.2 × 10^−05^	5.82 × 10^01^	6.34 × 10^00^
*C*_7_	7.25 × 10^01^	1.17 × 10^01^	5.43 × 10^01^	9.85 × 10^00^	5.64 × 10^01^	5.68 × 10^00^	7.56 × 10^01^	5.13 × 10^01^	4.67 × 10^01^	3.46 × 10^00^	9.23 × 10^02^	3.12 × 10^02^
*C*_8_	2.39 × 10^01^	2.94 × 10^01^	2.42 × 10^01^	4.48 × 10^00^	2.62 × 10^01^	3.75 × 10^00^	3.17 × 10^01^	1.44 × 10^01^	1.64 × 10^01^	4.36 × 10^00^	2.08 × 10^01^	3.99 × 10^00^
*C*_9_	0.00 × 10^00^	0.00 × 10^00^	0.00 × 10^00^	0.00 × 10^00^	0.00 × 10^00^	0.00 × 10^00^	0.00 × 10^00^	0.00 × 10^00^	6.3 × 10^−02^	1.4 × 10^−01^	5.49 × 10^03^	1.52 × 10^03^
*C*_10_	3.15 × 10^03^	6.19 × 10^02^	7.28 × 10^03^	3.28 × 10^02^	1.86 × 10^03^	2.83 × 10^02^	3.85 × 10^03^	1.92 × 10^03^	1.65 × 10^03^	3.85 × 10^02^	3.81 × 10^03^	4.74 × 10^02^
*C*_11_	1.49 × 10^01^	2.51 × 10^01^	1.67 × 10^01^	2.01 × 10^01^	2.00 × 10^01^	7.31 × 10^00^	1.08 × 10^01^	2.31 × 10^00^	2.22 × 10^01^	1.59 × 10^01^	1.95 × 10^02^	4.82 × 10^01^
*C*_12_	1.32 × 10^04^	1.62 × 10^04^	1.39 × 10^04^	8.83 × 10^03^	1.35 × 10^03^	9.05 × 10^02^	8.54 × 10^03^	9.68 × 10^03^	1.18 × 10^03^	3.99 × 10^02^	1.12 × 10^03^	3.74 × 10^02^
*C*_13_	2.42 × 10^01^	8.11 × 10^00^	2.95 × 10^01^	5.50 × 10^00^	3.11 × 10^01^	9.70 × 10^00^	2.71 × 10^01^	1.13 × 10^01^	3.99 × 10^01^	1.86 × 10^01^	3.99 × 10^02^	1.75 × 10^02^
*C*_14_	2.06 × 10^01^	1.46 × 10^01^	2.38 × 10^01^	6.04 × 10^00^	1.46 × 10^03^	3.03 × 10^03^	2.02 × 10^01^	1.01 × 10^01^	2.96 × 10^01^	3.03 × 10^00^	1.93 × 10^02^	5.62 × 10^01^
*C*_15_	6.54 × 10^00^	4.11 × 10^00^	7.10 × 10^00^	2.32 × 10^00^	3.49 × 10^02^	9.92 × 10^02^	8.18 × 10^00^	3.66 × 10^00^	3.73 × 10^01^	3.27 × 10^01^	2.14 × 10^02^	8.74 × 10^01^
*C*_16_	3.28 × 10^02^	4.08 × 10^02^	1.59 × 10^01^	9.80 × 10^00^	4.68 × 10^02^	1.60 × 10^02^	4.49 × 10^02^	5.06 × 10^02^	4.10 × 10^02^	1.27 × 10^02^	1.47 × 10^03^	4.66 × 10^02^
*C*_17_	2.46 × 10^02^	7.48 × 10^01^	2.71 × 10^01^	2.90 × 10^00^	7.38 × 10^01^	4.16 × 10^01^	1.02 × 10^02^	7.04 × 10^01^	5.13 × 10^01^	1.24 × 10^01^	8.69 × 10^02^	2.63 × 10^02^
*C*_18_	2.43 × 10^01^	1.07 × 10^00^	2.80 × 10^01^	8.32 × 10^00^	6.85 × 10^01^	4.16 × 10^01^	3.24 × 10^01^	1.59 × 10^01^	5.82 × 10^01^	4.37 × 10^01^	1.59 × 10^02^	7.48 × 10^01^
*C*_19_	4.11 × 10^00^	2.20 × 10^00^	5.61 × 10^00^	1.78 × 10^00^	2.49 × 10^01^	2.63 × 10^01^	7.37 × 10^00^	3.08 × 10^00^	1.20 × 10^01^	3.68 × 10^00^	5.91 × 10^02^	3.57 × 10^02^
*C*_20_	2.72 × 10^01^	5.15 × 10^01^	2.02 × 10^01^	7.15 × 10^00^	1.06 × 10^02^	5.03 × 10^01^	6.93 × 10^01^	9.83 × 10^01^	5.75 × 10^01^	3.66 × 10^01^	6.80 × 10^02^	1.94 × 10^02^
*C*_21_	2.45 × 10^02^	1.34 × 10^01^	2.21 × 10^02^	4.23 × 10^00^	2.26 × 10^02^	5.65 × 10^00^	2.25 × 10^02^	9.81 × 10^00^	2.17 × 10^02^	1.56 × 10^00^	4.15 × 10^02^	3.20 × 10^01^
*C*_22_	1.00 × 10^02^	0.00 × 10^00^	1.00 × 10^02^	0.00 × 10^00^	1.00 × 10^02^	0.00 × 10^00^	1.00 × 10^02^	0.00 × 10^00^	1.00 × 10^02^	0.00 × 10^00^	1.33 × 10^03^	1.96 × 10^03^
*C*_23_	3.76 × 10^02^	8.45 × 10^00^	3.61 × 10^02^	8.74 × 10^00^	3.72 × 10^02^	4.62 × 10^00^	3.76 × 10^02^	1.45 × 10^01^	3.65 × 10^02^	6.99 × 10^00^	7.97 × 10^02^	8.41 × 10^01^
*C*_24_	4.65 × 10^02^	1.15 × 10^01^	4.41 × 10^02^	4.84 × 10^00^	4.40 × 10^02^	4.80 × 10^00^	4.51 × 10^02^	1.83 × 10^01^	4.36 × 10^02^	2.58 × 10^00^	9.60 × 10^02^	7.35 × 10^01^
*C*_25_	3.87 × 10^02^	1.1 × 10^−01^	3.87 × 10^02^	2.7 × 10^−02^	3.87 × 10^02^	1.8 × 10^−01^	4.51 × 10^02^	1.83 × 10^01^	3.87 × 10^02^	3.3 × 10^−01^	3.95 × 10^02^	1.85 × 10^01^
*C*_26_	1.38 × 10^03^	1.52 × 10^02^	9.77 × 10^02^	7.79 × 10^01^	1.20 × 10^03^	2.89 × 10^01^	1.23 × 10^03^	9.75 × 10^01^	1.10 × 10^03^	7.06 × 10^01^	4.42 × 10^03^	1.14 × 10^03^
*C*_27_	5.02 × 10^02^	6.51 × 10^00^	4.94 × 10^02^	1.16 × 10^01^	5.04 × 10^02^	1.10 × 10^01^	5.01 × 10^02^	7.98 × 10^00^	5.06 × 10^02^	6.86 × 10^00^	7.59 × 10^02^	1.24 × 10^02^
*C*_28_	3.42 × 10^02^	7.91 × 10^01^	3.36 × 10^02^	5.35 × 10^01^	3.54 × 10^02^	5.68 × 10^01^	3.48 × 10^02^	7.37 × 10^01^	3.43 × 10^02^	5.62 × 10^01^	3.31 × 10^02^	5.81 × 10^01^
*C*_29_	4.19 × 10^02^	1.13 × 10^02^	4.23 × 10^02^	2.79 × 10^01^	4.86 × 10^02^	5.07 × 10^01^	4.61 × 10^02^	8.08 × 10^01^	4.69 × 10^02^	3.85 × 10^01^	1.56 × 10^03^	4.15 × 10^02^
*C*_30_	2.04 × 10^03^	1.35 × 10^02^	2.07 × 10^03^	4.59 × 10^01^	2.18 × 10^03^	1.69 × 10^02^	2.10 × 10^03^	1.06 × 10^02^	2.11 × 10^03^	7.53 × 10^01^	4.35 × 10^03^	1.43 × 10^03^
CPU time (s)		146.2		165.4		172.9		144.5		148.1		168.2
*w/l/t*				*13/11/5*		*14/10/5*		*16/9/4*		*14/11/4*		*25/4/0*
*p-values*				*0.839 ^=^*		*0.541 ^=^*		*0.030 ^+^*		*0.690 ^=^*		*0.001 ^+^*

‘+’ and ‘=’ stand for significantly better and equal, respectively.

**Table 5:** Comparison of IDEBW with other meta-heuristics on CEC-2017 functions.

*Fun*	IDEBW		EJaya		HMRFO		AGBSO		DisGSA		TDSD	
	Mean	SD	Mean	SD	Mean	SD	Mean	SD	Mean	SD	Mean	SD
***C*_1_**	**0.00** **× 10^00^**	0.00 × 10^00^	1.28 × 10^02^	2.03 × 10^02^	2.98 × 10^03^	2.34 × 10^03^	2.16 × 10^03^	2.63 × 10^03^	2.44 × 10^03^	1.17 × 10^03^	1.75 × 10^03^	9.03 × 10^02^
***C*_3_**	1.8 × 10^−08^	1.9 × 10^−07^	**4.9** × **10^−10^**	5.13 × 10^05^	6.14 × 10^01^	3.57 × 10^01^	4.97 × 10^01^	1.07 × 10^02^	4.93 × 10^03^	2.12 × 10^03^	4.04 × 10^04^	9.54 × 10^03^
***C*_4_**	5.86 × 10^01^	0.00 × 10^00^	2.64 × 10^01^	1.42 × 10^01^	3.64 × 10^01^	3.50 × 10^01^	9.02 × 10^01^	1.56 × 10^01^	1.02 × 10^02^	2.38 × 10^01^	**2.02 × 10^01^**	2.10 × 10^01^
***C*_5_**	3.55 × 10^01^	1.22 × 10^01^	5.21 × 10^01^	2.05 × 10^01^	6.00 × 10^01^	1.91 × 10^01^	**1.67 × 10^01^**	6.08 × 10^00^	1.75 × 10^01^	6.51 × 10^00^	7.99 × 10^01^	1.14 × 10^01^
***C*_6_**	**0.00 × 10^00^**	0.00 × 10^00^	4.17 × 10^00^	1.35 × 10^01^	4.9 × 10^−01^	1.07 × 10^00^	7.7 × 10^−05^	4.7 × 10^−05^	4.1 × 10^−05^	7.3 × 10^−05^	3.30 × 10^00^	6.9 × 10^−01^
***C*_7_**	7.25 × 10^01^	1.17 × 10^01^	1.10 × 10^02^	4.52 × 10^00^	1.17 × 10^02^	3.98 × 10^01^	5.10 × 10^01^	7.47 × 10^00^	**5.02 × 10^01^**	3.97 × 10^00^	1.32 × 10^02^	1.34 × 10^01^
***C*_8_**	2.39 × 10^01^	2.94 × 10^01^	7.34 × 10^01^	9.85 × 10^00^	6.53 × 10^01^	1.89 × 10^01^	**1.47 × 10^01^**	4.97 × 10^00^	1.71 × 10^01^	3.25 × 10^00^	8.33 × 10^01^	8.19 × 10^00^
***C*_9_**	**0.00 × 10^00^**	0.00 × 10^00^	2.30 × 10^02^	2.88 × 10^01^	4.64 × 10^01^	4.07 × 10^01^	**0.00 × 10^00^**	0.00 × 10^00^	1.8 × 10^−13^	6.2 × 10^−14^	1.71 × 10^03^	3.44 × 10^02^
***C*_10_**	3.15 × 10^03^	6.19 × 10^02^	3.82 × 10^03^	2.87 × 10^02^	3.47 × 10^03^	6.76 × 10^02^	**4.80 × 10^02^**	2.70 × 10^02^	1.98 × 10^03^	5.54 × 10^02^	2.19 × 10^03^	2.20 × 10^02^
***C*_11_**	**1.49 × 10^01^**	2.51 × 10^01^	8.64 × 10^01^	1.25 × 10^01^	4.29 × 10^01^	1.05 × 10^01^	5.07 × 10^01^	2.82 × 10^01^	9.61 × 10^01^	2.82 × 10^01^	6.86 × 10^01^	2.40 × 10^02^
***C*_12_**	1.32 × 10^04^	1.62 × 10^04^	**7.48 × 10^03^**	2.03 × 10^05^	3.65 × 10^04^	1.35 × 10^04^	6.12 × 10^05^	3.01 × 10^05^	9.75 × 10^03^	1.76 × 10^03^	2.91 × 10^05^	1.73 × 10^05^
***C*_13_**	**2.42 × 10^01^**	8.11 × 10^00^	2.56 × 10^03^	2.54 × 10^03^	1.48 × 10^04^	1.02 × 10^04^	1.07 × 10^04^	6.58 × 10^03^	4.75 × 10^03^	2.51 × 10^03^	6.95 × 10^02^	3.24 × 10^02^
***C*_14_**	**2.06 × 10^01^**	1.46 × 10^01^	1.12 × 10^02^	1.42 × 10^03^	1.68 × 10^03^	9.51 × 10^02^	2.74 × 10^03^	2.96 × 10^03^	3.41 × 10^03^	2.51 × 10^03^	8.42 × 10^03^	6.07 × 10^03^
***C*_15_**	**6.54 × 10^00^**	4.11 × 10^00^	9.58 × 10^02^	8.67 × 10^00^	2.87 × 10^03^	3.81 × 10^03^	3.47 × 10^03^	3.80 × 10^03^	1.66 × 10^03^	1.66 × 10^03^	3.56 × 10^02^	2.24 × 10^02^
***C*_16_**	3.28 × 10^02^	4.08 × 10^02^	4.74 × 10^02^	1.37 × 10^02^	6.30 × 10^02^	2.92 × 10^02^	**1.13 × 10^02^**	9.81 × 10^01^	5.71 × 10^02^	2.39 × 10^02^	4.85 × 10^02^	1.16 × 10^02^
***C*_17_**	2.46 × 10^02^	7.48 × 10^01^	1.19 × 10^02^	6.91 × 10^01^	1.95 × 10^02^	1.36 × 10^02^	**5.10 × 10^01^**	3.85 × 10^01^	1.71 × 10^02^	1.34 × 10^02^	9.38 × 10^01^	3.88 × 10^01^
***C*_18_**	**2.43 × 10^01^**	1.07 × 10^00^	4.04 × 10^03^	1.47 × 10^04^	8.15 × 10^04^	3.38 × 10^04^	9.27 × 10^04^	5.46 × 10^04^	4.13 × 10^04^	1.74 × 10^04^	8.14 × 10^04^	3.67 × 10^04^
***C*_19_**	**4.11 × 10^00^**	2.20 × 10^00^	2.54 × 10^02^	2.82 × 10^03^	2.52 × 10^03^	2.66 × 10^03^	5.39 × 10^03^	6.64 × 10^03^	3.67 × 10^03^	1.32 × 10^03^	1.53 × 10^02^	1.01 × 10^02^
***C*_20_**	**2.72 × 10^01^**	5.15 × 10^01^	3.27 × 10^02^	4.15 × 10^01^	2.70 × 10^02^	1.24 × 10^02^	1.01 × 10^02^	6.55 × 10^01^	1.74 × 10^02^	1.29 × 10^01^	1.38 × 10^02^	5.41 × 10^01^
***C*_21_**	2.45 × 10^02^	1.34 × 10^01^	2.51 × 10^02^	9.11 × 10^00^	2.52 × 10^02^	1.84 × 10^01^	**2.17 × 10^02^**	5.54 × 10^00^	2.28 × 10^02^	8.81 × 10^00^	2.22 × 10^02^	8.14 × 10^01^
***C*_22_**	**1.00 × 10^02^**	0.00 × 10^00^	**1.00 × 10^02^**	1.6 × 10^−06^	**1.00 × 10^02^**	2.4 × 10^−13^	**1.00 × 10^02^**	2.3 × 10^−06^	**1.00 × 10^02^**	4.7 × 10^−09^	1.11 × 10^02^	1.89 × 10^00^
***C*_23_**	3.76 × 10^02^	8.45 × 10^00^	4.18 × 10^02^	1.42 × 10^01^	4.30 × 10^02^	2.51 × 10^01^	**3.60 × 10^02^**	5.07 × 10^00^	3.73 × 10^02^	4.97 × 10^00^	4.54 × 10^02^	1.72 × 10^02^
***C*_24_**	4.65 × 10^02^	1.15 × 10^01^	4.89 × 10^02^	4.34 × 10^00^	4.81 × 10^02^	1.71 × 10^01^	4.36 × 10^02^	1.10 × 10^01^	**4.13 × 10^02^**	1.68 × 10^01^	4.25 × 10^02^	1.87 × 10^02^
***C*_25_**	**3.87 × 10^02^**	1.1 × 10^−01^	4.03 × 10^02^	8.94 × 10^00^	3.92 × 10^02^	1.37 × 10^01^	3.86 × 10^02^	1.11 × 10^00^	3.87 × 10^02^	2.11 × 10^00^	**3.83 × 10^02^**	1.15 × 10^01^
***C*_26_**	1.38 × 10^03^	1.52 × 10^02^	2.25 × 10^03^	5.46 × 10^02^	1.68 × 10^03^	8.49 × 10^02^	9.93 × 10^02^	7.67 × 10^01^	**2.00 × 10^02^**	1.8 × 10^−08^	2.21 × 10^02^	5.54 × 10^00^
***C*_27_**	**5.02 × 10^02^**	6.51 × 10^00^	5.54 × 10^02^	7.04 × 10^00^	5.45 × 10^02^	1.58 × 10^01^	5.05 × 10^02^	5.80 × 10^00^	5.48 × 10^02^	1.89 × 10^01^	5.21 × 10^02^	6.13 × 10^00^
***C*_28_**	3.42 × 10^02^	7.91 × 10^01^	3.80 × 10^02^	1.10 × 10^01^	3.34 × 10^02^	5.73 × 10^01^	3.80 × 10^02^	4.18 × 10^01^	3.66 × 10^02^	6.07 × 10^01^	4.09 × 10^02^	1.48 × 10^01^
***C*_29_**	**4.19 × 10^02^**	1.13 × 10^02^	6.21 × 10^02^	1.01 × 10^02^	7.66 × 10^02^	1.78 × 10^02^	4.67 × 10^02^	3.45 × 10^01^	6.35 × 10^02^	1.71 × 10^02^	5.90 × 10^02^	4.56 × 10^01^
***C*_30_**	**2.04 × 10^03^**	1.35 × 10^02^	4.88 × 10^03^	2.90 × 10^04^	3.91 × 10^03^	1.15 × 10^03^	5.14 × 10^04^	4.22 × 10^04^	5.17 × 10^03^	7.06 × 10^02^	5.04 × 10^03^	8.29 × 10^02^
**CPU time (s)**	146.2		**105.4**		165.2		154.4		159.2		189.3	
*w/l/t*			*24/4/1*		*25/3/1*		*16/11/2*		*17/10/2*		*22/7/0*	
p-value			*0.001 ^+^*		*0.001 ^+^*		*0.441 ^=^*		*0.248 ^=^*		*0.009 ^+^*	

‘+’ and ‘=’ stand for significantly better and equal, respectively.

**Table 6:** ‘Wilcoxon rank sum test’ outcomes for the CEC17 functions.

Algorithms		Pairwise Rank	ΣR^+^	ΣR^−^	z-Value	*p*-Value	Sig at α = 0.05
IDEBW vs.	TRADE	(1.47, 1.53)	127	173	0.657	0.511	=
	CJADE	(1.43, 1.57)	160	140	0.286	0.775	=
	DEGOS	(1.38,1.62)	191	133	0.794	0.427	=
	SHADE	(1.45, 1.55)	166	159	0.094	0.927	=
	IMODE	(1.14, 1.86)	392	43	3.773	0.001	+
	EJaya	(1.16, 1.84)	355	51	3.461	0.001	+
	HMRFO	(1.12, 1.88)	376	30	3.939	0.001	+
	AGBSO	(1.41, 1.59)	266	112	1.850	0.062	=
	DisGSA	(1.38, 1.62)	272	106	1.994	0.042	+
	TDSD	(1.24, 1.76)	356	79	2.995	0.003	+

‘+’, ‘−‘ and ‘=’ stand for significantly better, worst and equal, respectively.

**Table 7:** Friedman Ranks and Bonferroni–Dunn’s CD values for CEC17 functions.

DE Variants		Other Meta-Heuristics	
Algorithm	Rank	Algorithm	Rank
IDEBW	2.86	IDEBW	**2.31**
TRADE	**2.66**	EJAYA	3.95
CJADE	3.83	HMRFO	4.38
DEGOS	3.62	AGBSO	3.10
SHADE	2.97	DisGSA	3.50
IMODE	5.02	TDSD	3.76
CD (Level = 10%)	1.1428	CD (Level = 10%)	1.1428
CD (Level = 5%)	1.2656	CD (Level = 5%)	1.2656

**Table 8:** Performance evaluation of IDEBW on real-life optimization problems.

Problem	Iter.	Value	IDEBW	DEGOS	SHADE	CJADE	EJAYA	TDSD
RP_1_		Best	**0.00**	2.24 × 10^−20^	**0.00**	0.00	**1.400**	**0.00**
	600	Mean	**1.16**	3.11	1.82	2.2980	10.68	3.93
		SD	**0.9084**	6.95	2.60	6.1711	5.4506	4.97
		rank	**1**	5	2	3	6	4
RP_2_		Best	**0.5891**	0.7092	1.0345	0.7029	0.5000	0.8701
	2000	Mean	**0.7332**	1.467	1.2256	0.9171	1.0094	1.0234
		SD	**0.1924**	0.3537	0.0974	0.1066	0.3017	0.0773
		rank	**1**	5	2	3	6	4
RP_3_		Best	**13.770**	13.783	13.77	13.832	14.981	**13.77**
	100	Mean	**13.921**	14.362	14.28	14.329	15.006	13.93
		SD	**0.2856**	1.7475	0.20	1.212	2.302	0.17
		rank	**1**	5	3	4	6	2

## Data Availability

All related data is contained within the article.
